# Defining benefit threshold for extracorporeal membrane oxygenation in children with sepsis—a binational multicenter cohort study

**DOI:** 10.1186/s13054-019-2685-1

**Published:** 2019-12-30

**Authors:** Luregn J. Schlapbach, Roberto Chiletti, Lahn Straney, Marino Festa, Daniel Alexander, Warwick Butt, Graeme MacLaren, on behalf of the Australian & New Zealand Intensive Care Society (ANZICS) Centre for Outcomes & Resource Evaluation (CORE) and the Australian & New Zealand Intensive Care Society (ANZICS) Paediatric Study Group, Anusha Ganeshalingam, Claire Sherring, Simon Erickson, Samantha Barr, Andreas Schibler, Debbie Long, Luregn Schlapbach, Jan Alexander, Shane George, Gary Williams, Vicky Smith, Warwick Butt, Carmel Delzoppo, Johnny Millar, Ben Gelbart, Felix Oberender, Subodh Ganu, Georgia Letton, Jonathan Egan, Gail Harper, Marino Festa

**Affiliations:** 1 Paediatric Critical Care Research Group, Child Health Research Centre, The University of Queensland, Brisbane, Australia; 2 Paediatric Intensive Care Unit, Queensland Children’s Hospital, South Brisbane, QLD 4101 Australia; 3 Department of Pediatrics, Inselspital, Bern University Hospital, University of Bern, Bern, Switzerland; 4 University of Melbourne, Melbourne, Australia; 5 Paediatric Intensive Care Unit, The Royal Children’s Hospital, Melbourne, Australia; 6 Department of Epidemiology and Preventive Medicine, Monash University, Melbourne, Australia; 7 Paediatric Intensive Care Unit, Children’s Hospital Westmead, Sydney, Australia; 8 Kids Critical Care Research Group, Kids Research, Sydney Children’s Hospitals Network, Sydney, Australia; 9 Paediatric Intensive Care Unit, Perth Children’s Hospital, Perth, Australia; 10 Cardiothoracic Intensive Care Unit, National University Health System, Singapore, Singapore

**Keywords:** Childhood, Extracorporeal life support, Extracorporeal membrane oxygenation, Infection, Mortality, Pediatric, Prediction, Sepsis, Septic shock

## Abstract

**Background:**

The surviving sepsis campaign recommends consideration for extracorporeal membrane oxygenation (ECMO) in refractory septic shock. We aimed to define the benefit threshold of ECMO in pediatric septic shock.

**Methods:**

Retrospective binational multicenter cohort study of all ICUs contributing to the Australian and New Zealand Paediatric Intensive Care Registry. We included patients < 16 years admitted to ICU with sepsis and septic shock between 2002 and 2016. Sepsis-specific risk-adjusted models to establish ECMO benefit thresholds with mortality as the primary outcome were performed. Models were based on clinical variables available early after admission to ICU. Multivariate analyses were performed to identify predictors of survival in children treated with ECMO.

**Results:**

Five thousand sixty-two children with sepsis and septic shock met eligibility criteria, of which 80 (1.6%) were treated with veno-arterial ECMO. A model based on 12 clinical variables predicted mortality with an AUROC of 0.879 (95% CI 0.864–0.895). The benefit threshold was calculated as 47.1% predicted risk of mortality. The observed mortality for children treated with ECMO below the threshold was 41.8% (23 deaths), compared to a predicted mortality of 30.0% as per the baseline model (16.5 deaths; standardized mortality rate 1.40, 95% CI 0.89–2.09). Among patients above the benefit threshold, the observed mortality was 52.0% (13 deaths) compared to 68.2% as per the baseline model (16.5 deaths; standardized mortality rate 0.61, 95% CI 0.39–0.92). Multivariable analyses identified lower lactate, the absence of cardiac arrest prior to ECMO, and the central cannulation (OR 0.31, 95% CI 0.10–0.98, *p* = 0.046) as significant predictors of survival for those treated with VA-ECMO.

**Conclusions:**

This binational study demonstrates that a rapidly available sepsis mortality prediction model can define thresholds for survival benefit in children with septic shock considered for ECMO. Survival on ECMO was associated with central cannulation. Our findings suggest that a fully powered RCT on ECMO in sepsis is unlikely to be feasible.

## Background

Mortality in pediatric septic shock remains as high as 21% in pediatric intensive care units (PICU) [1–3]. In a substantial proportion of patients, shock will persist despite initial fluid resuscitation followed by vasoactive support as recommended by the Surviving Sepsis Campaign (SSC) [4] and the American College of Critical Care Medicine (ACCM) [5] guidelines. Refractory septic shock is characterized by profound circulatory dysfunction with alterations in myocardial function, vasoplegia, and failure of oxygen delivery to tissues, resulting in lactic acidosis [6, 7] and multi-organ failure, and accounts for most early sepsis deaths in children [8, 9].

Because extracorporeal membrane oxygenation (ECMO) can re-establish oxygen delivery in refractory shock, sepsis treatment guidelines have recommended consideration for its use as an adjunctive rescue therapy [4, 5]. This recommendation originated from case reports and retrospective series suggesting potential survival benefit [10–20]. However, these observational data were based on highly selected cohorts of single institutions and lacked adjustment for severity of illness. Currently, patient selection remains determined by individual and institutional practice rather than objective criteria. There is an unmet need for models defining the benefit threshold of ECMO in pediatric sepsis.

We have previously developed a pediatric sepsis score [21] in critically ill children with sepsis admitted to ICUs, which predicted sepsis mortality using a simple set of criteria available within 1 h of ICU admission. We aimed to adapt this approach to define risk-adjusted benefit thresholds for ECMO in sepsis. We hypothesized that rapid mortality prediction in pediatric sepsis can identify patients likely to benefit if treated with ECMO.

## Methods

We performed a multicenter binational retrospective cohort study of patients with sepsis and septic shock reported to the Australian and New Zealand Paediatric Intensive Care (ANZPIC) Registry [22]. The study was approved by the Human Research and Ethics Committee (Mater Health Services HREC, Brisbane, Australia) including waiver of informed consent. The ANZPIC registry prospectively records admissions of patients < 16 years to specialized PICUs and mixed ICUs in Australia and New Zealand [1].

### Inclusion criteria

Patients with age below 16 years that were non-electively admitted to a PICU or a general ICU in Australia and New Zealand between January 1, 2002, and December 31, 2016, with sepsis or septic shock at ICU admission were eligible. Patients were required to have sepsis or septic shock (including toxic shock) as the principal diagnosis, the underlying diagnosis of ICU admission, or as a high-risk diagnosis [23]. In addition, we included patients if they had any invasive infection (including meningitis, pneumonia/pneumonitis, peritonitis, necrotizing fasciitis, osteomyelitis, endocarditis, tracheitis, epiglottitis) as the principal or the underlying diagnosis and also had sepsis and/or septic shock (including toxic shock) in any other diagnostic field.

Controls were defined as patients < 16 years with sepsis and septic shock as defined above, which did not receive any ECMO.

We identified patients that underwent treatment with ECMO in the ANZPIC registry and manually checked these against the institutional ECMO databases of the six centers which provided ECMO during the study period. We checked the institutional ECMO databases and patient charts for the indications for ECMO and type of cannulation. Only veno-arterial (VA)-ECMO runs that were initiated to treat cardiovascular or combined cardiorespiratory failure in septic shock defined as per the 2005 International Pediatric Sepsis Consensus Conference [23] in patients who required inotropes prior to ECMO initiation and/or extracorporeal cardiopulmonary resuscitation (ECPR) were included as cases. Details on physiology upon initiation of ECMO, cannulation mode, and patient flow rates at 4 h post ECMO initiation were extracted from the institutional ECMO databases.

### Outcomes and definitions

The primary outcome was defined as ICU mortality. ICU mortality and ICU length of stay were available for 100% of patients. Patient comorbidities were extracted from the diagnostic coding in the registry as described elsewhere [1, 21]. The Pediatric Index of Mortality 2 (PIM2) [24] was used to assess patient illness severity at ICU admission.

### Statistics (Additional file 1)

Data are presented as percentages and numbers or medians with interquartile range (IQR). Two-sample Wilcoxon rank-sum (Mann-Whitney) tests were used to compare subgroups.

We previously demonstrated the high performance of a set of easily available clinical variables to predict sepsis-related mortality in critically ill children within 1 h of ICU admission [1]. We optimized this sepsis-specific mortality prediction model using a stepwise logistic regression approach in the dataset restricted to septic patients which were not treated with VA-ECMO, including additional variables on treatment delivered during admission. The mortality prediction model included patient characteristics (age, interhospital transfer, immunosuppression), physiological parameters (arterial hypotension, PaO_2_/FiO_2_ ratio, lactate), clinical characteristics (presence of shock on admission, dilated unresponsive pupils, cardiac arrest prior to admission), and treatment interventions (ventilation during the first hour of admission; intubation; continuous renal replacement therapy; high-frequency oscillation ventilation (HFOV), and inhaled nitric oxide). We defined arterial hypotension as systolic blood pressure below the 5th percentile for age and sex as previously described [25]. We used all variables significantly associated with the primary outcome in univariable analyses to develop the multivariable models. This “naive” baseline risk adjustment model was built using only those patients who did not receive ECMO as part of their treatment. Reverse stepwise regression was used to select final covariates with exit criteria of *p* < 0.2. We applied the Hosmer-Lemeshow goodness of fit test to assess calibration of the model in septic patients not treated with ECMO and described the area under the curve of receiver-operating-characteristic (AUROC) curve analysis. This disease-specific prediction model was then used for every patient (both septic controls and ECMO cases) to calculate the predicted mortality based on patient characteristics, severity upon presentation to intensive care, and level of support. We then used the linear prediction of the baseline risk adjustment model as a covariate in a second-stage model, the “treatment model,” to evaluate the effect of ECMO on ICU mortality for children with sepsis and septic shock. We estimated this second-stage model using a bootstrap procedure with 1000 repetitions. The samples were the same size as the total dataset and drawn with replacement from the original data stratified by ECMO treatment. The coefficients from each model repetition were used to estimate a distribution for the benefit threshold. The median was used to estimate the estimated threshold in baseline risk for benefit, and the 2.5th and 97.5th percentiles were used to estimate uncertainty intervals. This second-stage model was then repeated as a sensitivity analysis in only those patients coded with septic shock.

To analyze factors associated with survival of those children treated with ECMO for septic shock, we performed univariable followed by backward stepwise logistic regression including covariates with exit criteria of *p* < 0.2.

All analyses were conducted using Stata (version 15.0, Stata Corp, College Station, TX, USA).

## Results

### Cohort description

During the study period, 5062 children coded as sepsis and septic shock met the eligibility criteria, of which 80 (1.6%) were treated with ECMO for septic shock, which was confirmed using manual checking of patient records (Table 1). The crude mortality was 10.6% (483/4982) in controls and 45.0% (36/80) in ECMO cases (*p* < 0.001). The median time from ICU admission to death was 53.0 h (IQR 15.2–189.2, Additional file 2). In 61.3% (49/80) of ECMO cases and 45.2% (2250/4982) of controls, a bacterial pathogen was identified (Additional file 3). Patients treated with ECMO for septic shock were more likely to have undergone interhospital transfer and were sicker on admission to PICU as evidenced by lower systolic blood pressure, higher lactate, and higher PIM-2 scores. The proportion of children with sepsis treated with ECMO during the study period did not change substantially (14.2 ECMO-treated children per 1000 pediatric sepsis admissions in 2002–2009 compared with 17.0/1000 in 2010–2017, *p* = 0.425). In the ECMO group, 13 (16.3%) were diagnosed with ARDS, in comparison to 157 (3.2%) of patients in the control group (*p* < 0.001). Nine (11%) of the ECMO patients had undergone cannulation at a referring hospital.

**Table 1:** Baseline characteristics and severity characteristics in 5062 children admitted to intensive care units with sepsis and septic shock compared between children receiving extracorporeal membrane oxygenation (ECMO) and controls (no ECMO)

Characteristic	Variable	Sepsis or septic shock on admission—control group (no ECMO)	Sepsis or septic shock on admission—ECMO group	
		*N* = 4982	*n* = 80	*p* value^a^
Age	Age (days), median (IQR)	598.5 (103, 2588)	463 (44, 2546)	0.48
Age group	Infants (birth to 364 days)	2671 (53.6%)	45 (56.3%)	0.95
	1–4 years	915 (18.4%)	14 (17.5%)	
	5–9 years	874 (17.5%)	14 (17.5%)	
	10–15 years	522 (10.5%)	7 (8.8%)	
Indigenous status	Indigenous or Torres Strait Islander	411 (11.8%)	4 (6.0%)	0.14
Demographics	% male	2835 (56.9%)	49 (61.3%)	0.44
	Weight, median (IQR)	11.1 (5.2, 23.0)	10.55 (3.85, 25.0)	0.79
	Interhospital transfer	1846 (37.1%)	53 (66.3%)	< 0.001
Comorbidities	Congenital heart disease	288 (5.8%)	8 (10.0%)	0.11
	Immunosuppression^b^	801 (16.1%)	7 (8.8%)	0.076
	Oncology	733 (14.7%)	4 (5.0%)	0.015
Severity on PICU admission^c^	Shock on admission	2347 (47.1%)	72 (90.0%)	< 0.001
	Systolic blood pressure, median (IQR)	93 (80, 108)	85 (56, 100)	< 0.001
	Lactate, median (IQR)	3.26 (3.26, 3.26)	4.95 (3.26, 8.05)	< 0.001
	Dilated unresponsive pupils	45 (0.9%)	2 (2.5%)	0.14
	Cardiac arrest pre-admission	61 (1.2%)	13 (16.3%)	< 0.001
Therapy	Intubated	2537 (51.2%)	80 (100.0%)	< 0.001
	Mechanical ventilation in the first hour	2049 (41.2%)	68 (85.0%)	< 0.001
	Inotropes during 1st hour	1053 (30.0%)	57 (76.0%)	< 0.001
	Renal replacement	200 (4.0%)	41 (51.2%)	< 0.001
	High-frequency oscillation ventilation	180 (3.6%)	21 (26.3%)	< 0.001
	Inhaled nitric oxide	130 (2.6%)	23 (28.7%)	< 0.001
Outcome measures	PIM2, mean (SD)	0.08 (0.14)	0.25 (0.29)	< 0.001
	PIM2, median (IQR)	0.03 (0.01, 0.07)	0.13 (0.04, 0.35)	< 0.001
	Hospital mortality	501 (10.9%)	36 (45.0%)	< 0.001
	ICU mortality	415 (8.3%)	36 (45.0%)	< 0.001
	Mean PICU length of stay (hours), median (IQR)	56.65 (23.33, 135.77)	240.28 (59.28, 452.79)	< 0.001
Pathogen	Sum of patients with bacterial diagnosis	2250 (45.2%)	49 (61.3%)	0.004
	Viral coinfection	532 (10.7%)	10 (12.5%)	0.60
	No bacterial, fungal, or viral organism identified	2367 (47.5%)	25 (31.3%)	0.004

*PICU* pediatric intensive care unit, *PIM2* Pediatric Index of Mortality 2

^a^*p* value based on two-sample Wilcoxon rank-sum (Mann-Whitney) test

^b^Defined as either primary immunodeficiency or secondary immunodeficiency, including bone marrow transplants, oncology patients under active treatment, other solid organ transplant patients, and systemic immunosuppression such as for rheumatologic disease

^c^First observation, the measure must be obtained within the first 60 min of PICU admission

### Predicted sepsis mortality

The final multivariable model to predict ICU mortality in children with sepsis and septic shock admitted to ICU included severe respiratory failure (PaO2/FiO2 ratio, intubation, and treatment with HFOV); shock or cardiac arrest (arterial hypotension, shock on presentation, cardiac arrest pre ICU admission); metabolic (high lactate), central nervous system (dilated pupils), and renal (need for renal replacement) dysfunction; and underlying immunosuppression as significant predictors (Table 2). The model was well calibrated (Hosmer-Lemeshow goodness-of-fit test chi-square 9.83, *p* = 0.277) and predicted mortality with an AUROC of 0.879 (95% CI 0.864–0.895) which was significantly superior to mortality prediction using PIM-2 (AUROC 0.789; 0.765–0.813, *p* < 0.0001).

**Table 2:** Uni- and multivariable prediction models for ICU mortality in 5062 children admitted to intensive care units with sepsis and septic shock

Predictor variable		Univariable model		Multivariable model	
Group	Predictor	OR (95% CI)	*p* value	OR (95% CI)	*p* value
Respiratory	PaO2/FiO2 ratio	2.83 (2.34–3.43)	< 0.001	1.57 (1.28–1.91)	< 0.001
	Ventilation during the first hour	3.97 (3.15–4.96)	< 0.001	NS	
	Intubated	23.19 (14.58–36.89)	< 0.001	15.92 (9.76–25.96)	< 0.001
	High frequency ventilation	7.59 (5.50–10.46)	< 0.001	2.26 (1.48–3.45)	< 0.001
	Inhaled nitric oxide	7.15 (4.93–10.37)	< 0.001	NS	
Circulatory	SBP < 5th percentile	3.06 (2.45–3.82)	< 0.001	1.81 (1.39–2.36)	< 0.001
	Cardiac arrest pre-admission	6.50 (3.82–11.07)	< 0.001	2.36 (1.23–4.52)	0.01
	Shock on presentation	4.78 (3.73–6.07)	< 0.001	2.31 (1.76–3.04)	< 0.001
Metabolic	Lactate (mmol/l)	1.26 (1.20–1.32)	< 0.001	1.12 (1.07–1.18)	< 0.001
Renal	Renal replacement therapy	8.23 (6.06–11.17)	< 0.001	2.44 (1.72–3.47)	< 0.001
Neurologic	Dilated, unresponsive pupils	48.11 (23.00–11,062)	< 0.001	35.01 (15.35–79.84)	< 0.001
Patient	Immunosuppression	1.65 (1.29–2.10)	< 0.001	2.66 (1.95–3.63)	< 0.001

*OR* odds ratio, *CI* confidence interval, *SBP* systolic blood pressure

### Treatment benefit threshold

**Fig. 1:**
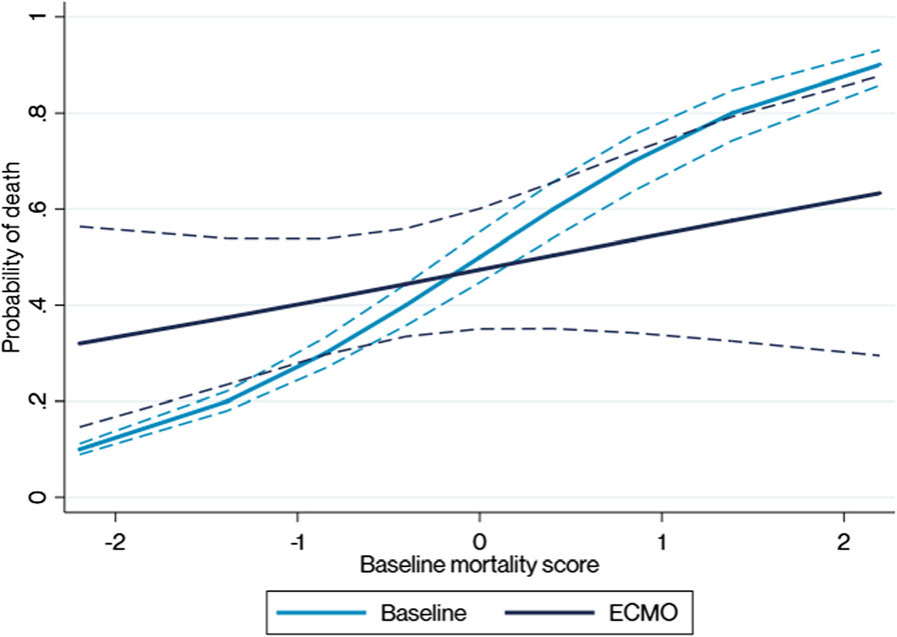
Estimating treatment benefit for children with sepsis and septic shock treated with extracorporeal life support (ECMO) versus controls. The marginal mean for estimated mortality is shown (y-scale) versus the baseline mortality score (x-scale) for children treated with ECMO (dark blue line) versus controls (light blue line). Full lines indicate the effect estimate, and dashed lines indicate 95% confidence intervals. The benefit threshold, defined as the baseline risk for which ECMO became beneficial, reflects the intersection of both lines at 47.1% predicted risk of mortality. The predicted mortality risk is adjusted for covariates on respiratory failure (PaO2/FiO2 ratio, intubation, treatment with HFOV); cardiovascular (arterial hypotension, shock on presentation, cardiac arrest pre ICU admission), metabolic (high lactate), central nervous system (dilated pupils), and renal (need for renal replacement) dysfunction; and underlying immunosuppression. The naïve baseline risk model is given by F1, where *p*_B_ is the baseline probability of mortality estimated among non-treated patients, *BRS* is the Baseline Risk Score, *B*_0_ is the intercept, and *B*_*n*_ and *X* represent a matrix of coefficients and risk factors. F1: *Logit*(*p*_*B*_) = *BRS* = *B*_0_ + *B*_*n*_*X*. The treatment model is given by F2, where *p*_D_ is the estimated mortality rate, *BRS* is the Baseline Risk Score (from F1), *B*_0_ is the intercept, and ECMO is a binary treatment variable (1 = yes). The final term is an interaction term between treatment and the baseline risk score. F2: Logit(*p*_D_) = *B*_0_ + *B*_1_ × *BRS* + *B*_2_ × *ECMO* + *B*_3_ × (ECMO ∗ BRS)

The benefit threshold, defined as the baseline mortality risk generated by our predictive model for which ECMO was associated with increased survival was calculated as 47.1% risk of mortality (95%CI 27.9–84.3%, Fig. 1 and Additional file 5). Of the children who received ECMO, 31.3% (25) had a baseline risk above the benefit threshold. The observed mortality for children treated with ECMO below the threshold was 41.8% (23 deaths), compared to a predicted mortality of 30.0% as per the baseline model (16.5 deaths; standardized mortality rate 1.40, 95% confidence interval 0.89–2.09). Among patients above the benefit threshold the observed mortality was 52.0% (13 deaths) compared to 68.2% as per the baseline model (16.5 deaths; standardized mortality rate 0.61, 95% confidence interval 0.39–0.92). Sensitivity analyses restricted to patients coded as septic shock in the ANZPICR resulted similarly (*n* = 80 cases, *n* = 2347 controls, benefit threshold of 47.7%; 95% CI 17.4–93.6%) (Additional file 6).

Based on these figures, we estimate that a future trial on ECMO in pediatric septic shock would require enrolment of at least 143 children treated with ECMO with baseline risk above the threshold to detect a mortality difference of 16.2% (52% versus 68.2%) with a power of 80% and a two-sided significance level of 0.05. Such a trial would need to screen approximatively 300,000 children admitted to ICU with sepsis and septic shock in our population.

### ECMO survivors versus ECMO fatal cases

Crude mortality in the 80 patients treated with VA-ECMO for septic shock was 45% (36/80) (Table 3 and Additional file 4). Thirty-two (40%) suffered a cardiac arrest prior to ECMO including 18 (23%) patients treated with ECPR for sepsis-related cardiac arrest. Fifty-seven out of 80 (71%) underwent primary open-chest cannulation in comparison to 23/80 (29%) who were initially cannulated peripherally. The median time from ICU admission to ECMO cannulation was 7 h in ECMO survivors compared to 2.8 h in ECMO deaths (*p* = 0.13). Children treated with ECMO who survived had lower admission lactate and PIM2 scores, higher pH and bicarbonate pre-ECMO, and a lower proportion with cardiac arrest prior to ECMO and were more often cannulated centrally. While the flow rates measured at 4 h were not significantly different between ECMO survivors and fatalities (Additional file 7), the mean flow rates of children cannulated centrally were 173 ml/kg/min in comparison to 129 ml/kg/min in children cannulated peripherally (*p* < 0.05). Multivariable analyses confirmed that higher lactate (odd’s ratio 1.21, 95%CI 1.04–1.40, *p* = 0.012), cardiac arrest prior to ECMO (OR 4.59, 1.58–13.10, *p* = 0.005) and central cannulation (OR 0.31, 0.10–0.98, *p* = 0.046) were significant independent predictors of mortality (Table 4, AUC 0.776).

**Table 3:** Extracorporeal membrane oxygenation (ECMO) run characteristics of 80 children with sepsis and septic shock compared between children who survived and children that died

Characteristic	Variable	Survived	Died	*p* value^a^
		*N* = 44 (55%)	*N* = 36 (45%)	
Age	Age (days), median (IQR)	1241 (36.5, 2595.5)	308.50 (63.5, 1272)	0.19
Severity on PICU admission^b^	Systolic blood pressure, median (IQR)	85 (58, 105)	77 (56, 96)	0.27
	Lactate, median (IQR)	4.15 (3.3, 6.2)	6.05 (3.3, 11.1)	0.002
	Dilated unresponsive pupils	1 (2%)	1 (3%)	0.89
	Cardiac arrest pre-admission	6 (14%)	7 (19%)	0.48
	PIM2, mean (SD)	0.19 (0.26)	0.32 (0.31)	0.052
	PIM2, median (IQR)	0.09 (0.04, 0.21)	0.22 (0.06, 0.51)	0.033
Therapy in PICU	Intubated	44 (100%)	36 (100%)	NA
	Mechanical ventilation during 1st hour	37 (84%)	31 (86%)	0.80
	Inotropes during 1st hour	27 (66%)	30 (88%)	0.055
	Renal replacement	27 (61%)	14 (39%)	0.045
	High-frequency oscillation ventilation	11 (25%)	10 (28%)	0.78
	Inhaled nitric oxide	14 (32%)	9 (25%)	0.50
Pre-ECMO physiology	CPR pre ECMO	11 (25%)	21 (58%)	0.002
	pH, median (IQR)	7.14 (7.06, 7.26)	7.04 (6.98, 7.19)	0.028
	pCO2, median (IQR)	47 (40, 57)	48 (37, 56)	0.67
	pO2, median (IQR)	58.5 (40.5, 111.5)	77 (43, 128)	0.53
	SaO2, median (IQR)	90 (71, 96)	94 (86, 98)	0.19
	HCO3, median (IQR)	17 (13, 23)	14 (9, 18)	0.027
	PIP/Amplitude, median (IQR)	30 (26, 38.5)	35 (26, 48)	0.27
	PEEP, median (IQR)	8 (6, 10)	10 (7, 10.5)	0.17
	MAP, median (IQR)	16.0 (13.6, 22.7)	19.0 (14.0, 26.0)	0.24
	Respiratory Rate/Hz, median (IQR)	20 (16, 30)	26.5 (8, 30)	0.68
	FiO2 (%), median (IQR)	100 (60, 100)	100 (92.5, 100)	0.45
	SBP, median (IQR)	80 (60, 100)	70 (53, 91)	0.56
	DBP, median (IQR)	40 (30, 45)	44 (30, 50)	0.42
	MBP, median (IQR)	51.5 (38, 60)	53.5 (38.5, 62)	0.96
ECMO category	Neonatal	12 (27%)	9 (25%)	0.82
	Pediatric	32 (73%)	27 (75%)	
	ECPR	6 (14%)	12 (33%)	< 0.001
ECMO support	Veno-arterial only	39 (89%)	35 (97%)	0.15
	Veno-arterial combined with venovenous	5 (11%)	1 (3%)	
	Time to ECMO (hours)^**c**^	7.00 (1.41, 45.00)	2.81 (1.00, 13.68)	0.13
	Central cannulation (initial)	35 (80%)	22 (61%)	0.070
	ECMO flow day 1 (ml/kg/min), median (IQR)	161 (134, 194)	156 (101, 190)	0.51
	ECMO support duration (days), median (IQR)	4.9 (3.7, 8.2)	1.7 (0.5, 7.1)	< 0.001
	PICU length of stay (days), median (IQR)	13.0 (8.9, 28.7)	2.3 (0.8, 10.9)	< 0.001

*CPR* cardiopulmonary resuscitation, *DBT* diastolic arterial blood pressure, *ECMO* extracorporeal life support, *ECPR* extracorporeal cardiopulmonary resuscitation, *MAP* mean airway pressure, *MBP* mean arterial blood pressure, *PEEP* positive end-expiratory pressure, *PICU* pediatric intensive care unit, *PIM2* Pediatric Index of Mortality 2, *PIP* peak inspiratory pressure, *SBP* systolic arterial blood pressure

^a^*p* value based on two-sample Wilcoxon rank-sum (Mann-Whitney) test

^b^First observation, the measure must be obtained within the first 60 min of PICU admission

^c^Time from PICU admission to ECMO cannulation

**Table 4:** Uni- and multivariable prediction models for ICU mortality in 80 children treated with extracorporeal membrane oxygenation for septic shock

Predictor variable		Univariable model		Multivariable model	
Group	Predictor	OR (95% CI)	*p* value	OR (95% CI)	*p* value
Metabolic	Lactate (mmol/l)	1.21 (1.06–1.38)	0.004	1.21 (1.04–1.40)	0.012
	pH pre ECMO	0.02 (0.00–0.64)	0.026	NS	
	HCO3- pre ECMO	0.88 (− 0.79 to 0.98)	0.017	NS	
Circulatory	Cardiac arrest pre-ECMO	4.20 (1.62–10.87)	0.003	4.59 (1.58–13.10)	0.005
Severity	Pediatric Index of Mortality-2	1.24 (0.98–1.57)	0.079	NS	
ECMO delivery	Central cannulation	0.40 (0.15–1.09)	0.074	0.31 (0.10–0.98)	0.046
	Flow at 4 h in ml/kg/min	1.00 (0.99–1.00)	0.271	NS	

*OR* odds ratio, *CI* confidence interval, *SBP* systolic blood pressure

## Discussion

Up to 50% of pediatric sepsis deaths occur within the first 24 h of admission [8, 21, 26, 27], predominantly because of refractory shock with circulatory failure. Any hypothetical novel intervention in sepsis would need to be applied within a few hours of PICU admission, and result in rapid physiologic improvement to yield any chance of major survival benefit. Such pharmacological interventions are not in sight; in contrast, mechanical circulatory support can be provided within a short time frame and can result in immediate improvement of circulatory status, but exposes patients to substantial, potentially life-threatening side effects. In this binational cohort including critically ill children with sepsis and septic shock, we demonstrated that a mortality prediction model based on 12 clinical variables allows discrimination of patients more likely to have treatment benefit from VA-ECMO therapy. To the best of our knowledge, this is the first study to assess benefit threshold of ECMO in sepsis, and the largest and only population-based study to report on risk-adjusted outcomes of ECMO in pediatric sepsis. Multivariable analyses identified lower lactate, absence of a cardiac arrest prior to ECMO, and central cannulation as independent protective factors for survival in children treated with ECMO for septic shock.

We identified a predicted mortality of 47.1% as the threshold above which ECMO was likely beneficial for children with septic shock. In children with lower disease-specific predicted mortality, the potential for harm may outweigh benefits related to ECMO. ECMO was initiated within the first few hours of PICU admission in the majority of children with refractory septic shock, which supports the need for rapid outcome prediction based on a set of clinical parameters that can be assessed within the first hour of admission to PICU. Recent studies have highlighted the promise of relatively simple clinical tools to assist in prediction of ECMO treatment benefit versus expected mortality in patients with acute respiratory distress syndrome [28, 29]. Importantly, our model incorporates several variables that were identified as essential criteria for pediatric refractory septic shock, specifically lactate, and severe cardiovascular dysfunction [9]. Our model predicting sepsis mortality with an AUC of 0.879 was developed from the previously published sepsis score [21], which permitted much better disease-specific risk prediction that commonly used scores such as PIM or PRISM.

Refractory septic shock in neonates and children carries a very high mortality as demonstrated by a recent multicenter study [9], and survivors often suffer from disability related to limb loss and neurocognitive impairment [2, 9, 30]. While current sepsis treatment guidelines recommend use of ECMO as an adjunctive rescue therapy, these recommendations are based on highly selected small single-center reports which hinder generalizability [10, 12, 14, 31]. In view of the rapidly expanding use of ECMO and an increasing number of adult and pediatric ECMO centers, there is an urgent need to understand the value of this therapy in sepsis. We here demonstrate proof of concept of a sepsis-specific survival prediction model which can enable appropriate resource use by early identification of patients most likely to benefit from ECMO. In addition, the model allows risk-adjusted comparison of ECMO outcomes for the purpose of benchmarking and quality control.

The largest series to date on children treated with ECMO for sepsis was based on the US Pediatric Health Information System database and suggested a significant increase in use of ECMO in recent years [32]. The mortality in this study was 47.8% for children who received any form of ECMO, which is comparable to our findings restricted to VA-ECMO for septic shock. While the US study was based on data from 43 PICUs participating in the Children’s Hospital Association, representing approximatively 15% of US PICUs, our study was based on the Australian and New Zealand prospective pediatric ICU registry which captures admissions to all PICUs, including 100% of pediatric ECMO centers. A recent study assessed retrospective data on 164 children admitted to 7 PICUs in 5 countries with septic shock, including 44 VA-ECMO runs, and observed a reduced crude mortality in the subgroup of patients with cardiac arrest treated with ECMO [33]. Multivariable analyses identified high lactate and cardiac arrest as significant mortality predictors, and a possible association between higher ECMO flow rates and improved survival. This supports previous single-center series [10, 31, 34] suggesting substantial mortality reduction when using a protocolized approach including central cannulation with larger size cannulae to achieve higher ECMO flow rates, compared to historic controls, with survival to discharge as high as 74%. The major limitation in these previous studies on ECMO in pediatric sepsis is the lack of risk-adjustment and failure to control for confounding by severity and indication.

Further validation in independent cohort is required to address several limitations of this study: First, ECMO and disease-specific outcomes may vary from site to site resulting in variable thresholds for treatment benefit. In addition, the use of central cannulation in non-cardiac surgery patients is something that may be more commonly used in Australian PICUs. Second, the study spanned across 15 years, and patient population, microbiology, and thresholds to initiate treatment may have changed [35]. While classic meningococcal shock associated with purpura fulminans has become rare, current septic shock phenotypes are often characterized by difficult source control, hypercoagulopathy, necrotizing pneumonia, and challenges related to host comorbidities including immunosuppression [36, 37]. Third, despite the fact that this is the largest study in the field, the inclusion of only 80 ECMO patients resulted in wide confidence intervals on treatment benefit thresholds. Fourth, due to the rapid dynamics of septic shock, repeat characterization and trend analysis of patient physiology several hours after PICU admission rather than within the first hour of PICU admission may more accurately reflect real time decision-making and may possibly result in even higher discriminatory performance of the prediction tool. We acknowledge that a proportion of patients managed on ECMO for sepsis had undergone prior interhospital transfer, implying that the true duration from hospital admission to cannulation was underestimated in some. Fifth, while we manually checked the phenotype of ECMO cases against medical records to ensure criteria for shock, including inotrope treatment, were met prior to ECMO, the prospective databases accessed were not designed to investigate refractory septic shock. We did not have information available on pre-PICU treatment, including delays in intravenous antimicrobial therapy [38, 39], and timing of inotrope initiation. Sixth, we assessed all-cause mortality and did not analyze cause of death or underlying diseases, which may affect decisions to stop treatment in this patient group. Seventh, information on individual inotrope doses was not available, which potentially may have yielded additional discriminatory value as recently demonstrated in a multicenter study on refractory septic shock [9]. Finally, we did not collect data on long-term cognitive and behavioral outcomes which may be severely affected in sepsis survivors [40].

## Conclusions

This large binational study indicates that ECMO is used in approximatively 1 out of 60 children admitted to PICU with sepsis and septic shock. We were able to demonstrate thresholds for survival benefit of ECMO using an easily available sepsis mortality prediction model. In our cohort, ECMO was unlikely to confer a survival benefit for children with sepsis and septic shock with below 47% predicted mortality. Our findings demonstrate that, given the rarity of ECMO therapy for septic shock at population level, a sufficiently powered randomized controlled trial to demonstrate ECMO survival benefit is unlikely to be feasible. Instead, future research should seek to enhance the precision of individual treatment benefit prediction through sequential assessment of the score. Applying dynamic information which integrates response to therapy during the first hours of ICU support will be of value as a further criterion to identify a trajectory towards cardiac arrest and death. In addition, future studies should test the application of our risk score for the purpose for benchmarking of mortality outcomes, quality improvement, and consideration for enrolment in trials of novel adjunctive therapies such as extracorporeal-based anti-inflammatory interventions [41].

## Supplementary information

**Additional file 1.** Supplementary Methods.

**Additional file 2. **Kaplan-Meier time to death curve is shown (*n*=537, panel C) out of 5,062 children admitted to ICU with sepsis/septic shock.

**Additional file 3.** Pathogens identified in 5,062 children admitted to Intensive Care Units with sepsis and septic shock compared between children receiving veno-arterial Extracorporeal Membrane Oxygenation (ECMO) and controls (no ECMO).

**Additional file 4.** Demographic and microbiological characteristics of 80 children with sepsis and septic shock treated with veno-arterial Extracorporeal Membrane Oxygenation (ECMO) compared between children who survived and children that died.

**Additional file 5.** Distribution of predicted mortality.

**Additional file 6. **Sensitivity analysis to estimating treatment benefit for children with septic shock treated with Extracorporeal life support (ECMO, *n*=80) versus controls (*n*=2347), restricted to patients coded as septic shock in the ANZPIC registry.

**Additional file 7. **Extracorporeal Membrane Oxygenation (ECMO) flow rates measured at 4 hours (in ml/kg/min) post cannulation in *n*=80 children treated with veno-arterial ECMO for septic shock. Flow rates are compared between children that underwent peripheral cannulation (*n*=23) versus children that underwent central cannulation (*n*=57), split into those that survived (*n*=44) and that died (*n*=36).

## Data Availability

The data that support the findings of this study are available from the Australian and New Zealand Paediatric Intensive Care (ANZPIC) Registry, but restrictions apply to the availability of these data, which were used under license for the current study, and so are not publicly available.

## Abbreviations

ECMO: Extracorporeal membrane oxygenation
OR: Odds ratio
PICU: Pediatric intensive care unit
PIM: Pediatric Index of Mortality
SSC: Surviving Sepsis Campaign
VA: Veno-arterial
VV: Venovenous
