# Supplementary material for: Defining benefit threshold for extracorporeal membrane oxygenation in children with sepsis—a binational multicenter cohort study
Source: Crit Care. 2019 Dec 30;23:429. doi: 10.1186/s13054-019-2685-1 (PMC6937937; doi:10.1186/s13054-019-2685-1)
Supplement: Supplementary file 1 — Additional file 1. Supplementary Methods. [file 13054_2019_2685_MOESM1_ESM.pdf]

Additional File 1: Supplementary Methods:

Overview

We performed a multicenter binational retrospective cohort study of patients below 16 years of age with sepsis and septic shock reported to the Australian and New Zealand Paediatric Intensive Care (ANZPIC) Registry[1]. The study was approved by the Human Research and Ethics Committee (Mater Health Services HREC, Brisbane, Australia) including waiver of informed consent.

The ANZPIC registry prospectively records admissions of patients <16 years to specialized PICUs and mixed ICUs in Australia and New Zealand. Data are audited biennially by independent auditors and central validation of data is performed. We extracted prospectively recorded data on baseline and demographic characteristics, diagnostic codes, severity upon presentation including physiological variables, severity markers, and outcomes as previously described[2]. Immunosuppression was defined as either primary immunodeficiency and secondary immunodeficiency, including bone marrow transplants, oncology patients including leukaemia, lymphoma and other tumors, and other solid organ transplant patients. Cardiac arrest was assessed both pre-ICU (in all patients, including ECMO cases and controls), and pre-ECMO (in ECMO cases).

Statistics:

Data are presented as percentages and numbers or means with standard deviation. Two-sample Wilcoxon rank-sum (Mann-Whitney) tests were used to compare subgroups. To analyse factors associated with survival of children treated with ECMO for septic shock, we performed univariable and then backward stepwise logistic regression including covariates with an exit criteria of $p<0.2$ in the dataset of $n=80$ patients treated with ECMO for sepsis. As ECMO flow was colinear with central cannulation it was dropped from the model.

We previously demonstrated the high performance of a set of easily available clinical covariates to predict sepsis-related mortality in critically ill children within one hour of ICU admission[2]. We optimized this sepsis-specific mortality prediction model using a stepwise logistic regression approach in the dataset restricted to septic patients which were not treated with VA-ECMO, including additional variables on treatment delivered during admission. The mortality prediction model included patient characteristics (age, interhospital transfer, immunosuppression), physiological parameters (arterial hypotension, PaO_2/FiO_2 ratio, lactate), clinical characteristics (presence of shock on admission, dilated unresponsive pupils, cardiac arrest prior to ICUn admission), and treatment interventions (ventilation during the first hour of admission defined as provision of either invasive or non-invasive ventilation; intubation; continuous renal replacement therapy; high-frequency oscillation ventilation (HFOV), and inhaled nitric oxide). In patients where no PaO_2/FiO_2 ratio was documented, we derived PaO_2/FiO_2 from SpO_2/FiO_2 ratio as previously described[3]. We defined arterial hypotension as systolic blood pressure below the 5th percentile for age and sex as previously described[4]. During the study period, mandatory collection of physiological parameters obtained within one hour of arrival in the ICU was performed, and the first observation was recorded. Missing data for lactate was imputed using mean imputation, and missing data for systolic blood pressure at the time of admission was imputed using age-specific mean imputation.

We used all variables significantly associated with the primary outcome in univariable analyses to develop the multivariable models. This ‘naive’ baseline risk adjustment model was built using only those patients who did not receive ECMO as part of their treatment. Reverse step wise regression was used to select final covariates with an exit criteria of $p<0.2$. We applied the Hosmer-Lemeshow goodness of fit test to assess calibration of the model in septic patients not treated with ECMO and described the Area under the Curve of Receiver-Operating-Characteristic curve analysis. This disease-specific prediction model was then used for every patient (both septic controls and ECMO cases) to calculate the predicted mortality based on patient characteristics, severity upon presentation to intensive care, and level of support. We then used the linear prediction of the baseline risk adjustment model as a covariate in a second stage model, the ‘treatment model’, to evaluate the effect of ECMO on ICU mortality for children with sepsis and septic shock. We estimated this second stage model using a bootstrap procedure with 1000 repetitions. The samples were the same size as the total dataset and drawn with replacement from the original data stratified by ECMO treatment. The coefficients from each model repetition were used to estimate a distribution for the benefit threshold. The median was used to estimate the estimated threshold in baseline risk for benefit and the 2.5^{th} and 97.5^{th} percentiles were used to estimate uncertainty intervals.

This second stage model was then repeated as a sensitivity analysis in only those patients coded with septic shock.

An interaction between the baseline linear prediction and the treatment variable (ECMO) was used to examine the potential for differential impacts of ECMO by baseline risk of mortality. We plotted the predicted mortality (marginal mean) for each over the linear prediction from the baseline model. All analyses were conducted using Stata (version 15.0, Stata Corp, College Station, Texas, USA).

References:

1. Straney L, Clements A, Parslow RC, Pearson G, Shann F, Alexander J, Slater A, (2013) Paediatric index of mortality 3: an updated model for predicting mortality in pediatric intensive care*. Pediatric critical care medicine : a journal of the Society of Critical Care Medicine and the World Federation of Pediatric Intensive and Critical Care Societies 14: 673-681
2. Schlapbach LJ, Straney L, Alexander J, MacLaren G, Festa M, Schibler A, Slater A, Group APS, (2015) Mortality related to invasive infections, sepsis, and septic shock in critically ill children in Australia and New Zealand, 2002-13: a multicentre retrospective cohort study. Lancet Infect Dis 15: 46-54
3. Khemani RG, Patel NR, Bart RD, 3rd, Newth CJ, (2009) Comparison of the pulse oximetric saturation/fraction of inspired oxygen ratio and the PaO2/fraction of inspired oxygen ratio in children. Chest 135: 662-668
4. Haque IU, Zaritsky AL, (2007) Analysis of the evidence for the lower limit of systolic and mean arterial pressure in children. Pediatr Crit Care Med 8: 138-144
