# Supplementary material for: Defining benefit threshold for extracorporeal membrane oxygenation in children with sepsis—a binational multicenter cohort study
Source: Crit Care. 2019 Dec 30;23:429. doi: 10.1186/s13054-019-2685-1 (PMC6937937; doi:10.1186/s13054-019-2685-1)

Additional File 2: Kaplan-Meier time to death curve is shown (n=537, panel C) are shown out of 5,062 children admitted to ICU with sepsis/septic shock. The median time from ICU admission to death was 53.0 hours (IQR: 15.2-189.2) across all patients and controls who died during hospital admission.

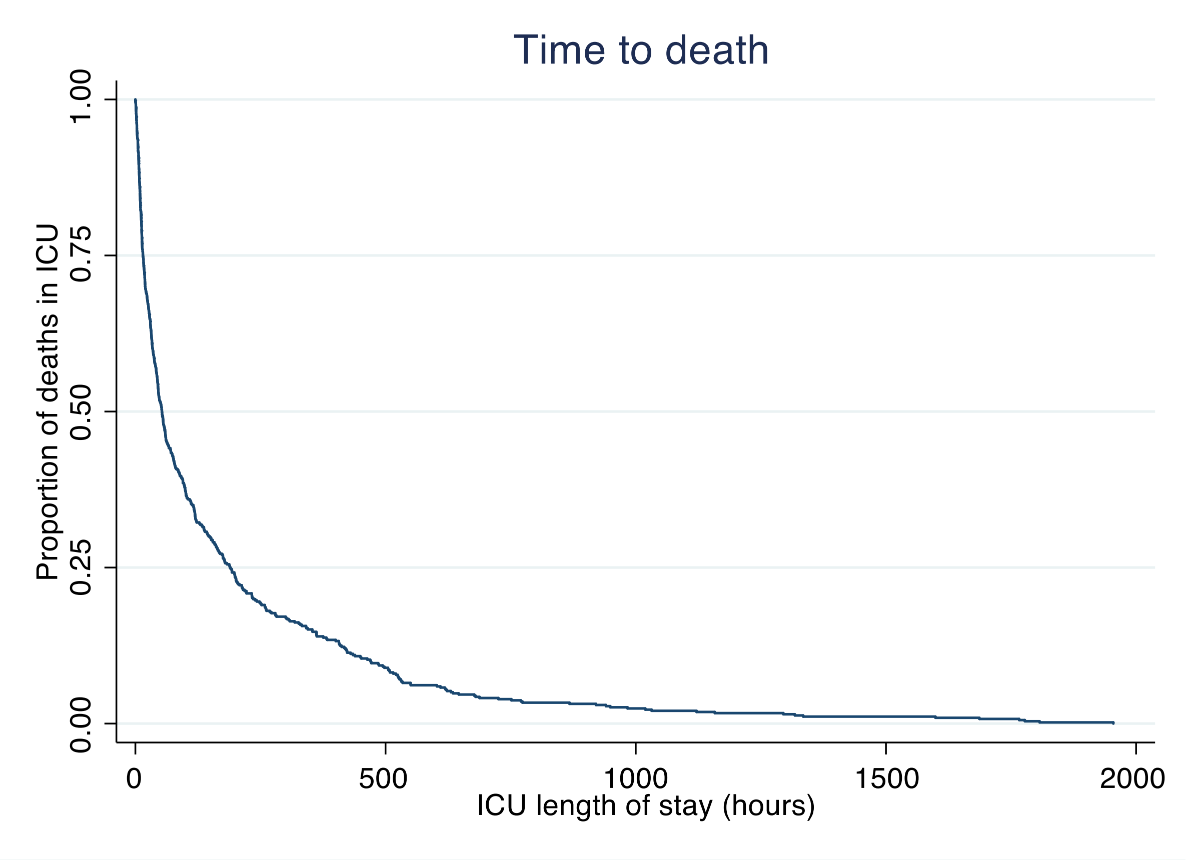

Supplement: Supplementary file 2 — Additional file 2. Kaplan-Meier time to death curve is shown (n=537, panel C) out of 5,062 children admitted to ICU with sepsis/septic shock. [file 13054_2019_2685_MOESM2_ESM.pdf]
