# Supplementary material for: Defining benefit threshold for extracorporeal membrane oxygenation in children with sepsis—a binational multicenter cohort study
Source: Crit Care. 2019 Dec 30;23:429. doi: 10.1186/s13054-019-2685-1 (PMC6937937; doi:10.1186/s13054-019-2685-1)
Supplement: Supplementary file 3 — Additional file 3. Pathogens identified in 5,062 children admitted to Intensive Care Units with sepsis and septic shock compared between children receiving veno-arterial Extracorporeal Membrane Oxygenation (ECMO) and controls (no ECMO). [file 13054_2019_2685_MOESM3_ESM.pdf]

Additional File 3: Pathogens identified in 5,062 children admitted to Intensive Care Units with sepsis and septic shock compared between children receiving veno-arterial Extracorporeal Membrane Oxygenation (ECMO) and controls (no ECMO).

		**Sepsis or septic shock on admission**		
		**No ECMO**	**ECMO group**	
Pathogen group	***Pathogen***	***N=4982***	***N=80***	***p-value***[a]
Bacteria	*N. meningitidis*	528 (10.6%)	10 (12.5%)	0.58
	Group B streptococcus	118 (2.4%)	3 (3.8%)	0.42
	Group A Streptococcus, viridans streptococcus	271 (5.4%)	13 (16.3%)	<0.001
	S. pneumoniae	133 (2.7%)	1 (1.3%)	0.43
	S. aureus	494 (9.9%)	12 (15.0%)	0.13
	E. coli	202 (4.1%)	2 (2.5%)	0.48
	Pseudomonas aeruginosa	115 (2.3%)	3 (3.8%)	0.40
	Klebsiella sp.	98 (2.0%)	1 (1.3%)	0.65
	H. influenzae	18 (0.4%)	0 (0.0%)	0.59
	Other bacteria	476 (9.6%)	7 (8.8%)	0.81
	Sum of patients with bacterial diagnosis	2250 (45.2%)	49 (61.3%)	0.004
Virus	Influenza viruses	93 (1.9%)	1 (1.3%)	0.69
	Parainfluenza viruses	63 (1.3%)	3 (3.8%)	0.052
	Respiratory Syncytial Virus	119 (2.4%)	2 (2.5%)	0.95
	Adenovirus	74 (1.5%)	1 (1.3%)	0.86
	Herpesviruses[b]	113 (2.3%)	3 (3.8%)	0.38
	Human Metapneumovirus	17 (0.3%)	1 (1.3%)	0.18
	Enterovirus	69 (1.4%)	0 (0.0%)	0.29

Other virus	27 (0.5%)	0 (0.0%)	0.51
Sum of patients with viral coinfection	532 (10.7%)	10 (12.5%)	0.60
No organism identified	2367 (47.5%)	25 (31.3%)	0.004

[a] p-value based on Two-sample Wilcoxon rank-sum (Mann-Whitney) test

[b] CMV, EBV, VZV
