# Supplementary material for: Defining benefit threshold for extracorporeal membrane oxygenation in children with sepsis—a binational multicenter cohort study
Source: Crit Care. 2019 Dec 30;23:429. doi: 10.1186/s13054-019-2685-1 (PMC6937937; doi:10.1186/s13054-019-2685-1)
Supplement: Supplementary file 4 — Additional file 4. Demographic and microbiological characteristics of 80 children with sepsis and septic shock treated with veno-arterial Extracorporeal Membrane Oxygenation (ECMO) compared between children who survived and children that died. [file 13054_2019_2685_MOESM4_ESM.pdf]

Additional File 4: Demographic and microbiological characteristics of 80 children with sepsis and septic shock treated with veno-arterial Extracorporeal Membrane Oxygenation (ECMO) compared between children who survived and children that died.

Characteristic	Variable	Survived N=44 (55%)	Died N=36 (45%)	*p-value*[a]
**Age**	age (days), median (IQR)	1241 (36.5, 2595.5)	308.50 (63.5, 1272)	0.19
	Infants (birth-364d)	20 (45%)	25 (69%)	0.068
	1-4 years	11 (25%)	3 (8%)	
	5-9 years	10 (23%)	4 (11%)	
	10-15 years	3 (7%)	4 (11%)	
**Indigenous status**	Indigenous or Torres Strait Islander	2 (6%)	2 (6%)	0.88
**Demographics**	% male	27 (61%)	22 (61%)	0.98
	Weight, median (IQR)	15.0 (3.55, 25.5)	8.3 (5.0, 18.0)	0.84
	Interhospital transfer	30 (68%)	23 (64%)	0.69
**Comorbidities**	Congenital heart disease	5 (11%)	3 (9%)	0.65
	Immunosuppression	2 (5%)	5 (14%)	0.14
	Oncology	2 (5%)	2 (6%)	0.84
**ARDS**	Acute Respiratory Distress Syndrome	8 (18%)	5 (14%)	0.61
**Year of admission**	2003	2 (5%)	1 (3%)	0.98
	2004	1 (2%)	1 (3%)	
	2005	1 (2%)	1 (3%)	
	2006	4 (9%)	2 (6%)	
	2007	4 (9%)	2 (6%)	
	2008	4 (9%)	1 (3%)	
	2009	2 (5%)	4 (11%)	

	2010	6 (14%)	4 (11%)	
	2011	4 (9%)	4 (11%)	
	2012	6 (14%)	4 (11%)	
	2013	3 (7%)	3 (8%)	
	2014	1 (2%)	2 (6%)	
	2015	2 (5%)	2 (6%)	
	2016	4 (9%)	5 (14%)	
Pathogen group	***Pathogen***			
Bacteria	*N. meningitidis*	7 (16%)	3 (8%)	0.31
	Group B streptococcus	3 (7%)	0 (0%)	0.11
	Group A Streptococcus, viridans streptococcus	10 (23%)	3 (8%)	0.083
	S. pneumoniae	0 (0%)	1 (3%)	0.27
	S. aureus	8 (18%)	4 (11%)	0.38
	E. coli	1 (2%)	1 (3%)	0.89
	Pseudomonas aeruginosa	1 (2%)	2 (6%)	0.44
	Klebsiellaspp.	1 (2%)	0 (0%)	0.36
	H.influenzae	0 (0%)	0 (0%)	NA
	Other bacteria	3 (7%)	4 (11%)	0.50
	Sum of patients with bacterial diagnosis	31 (70%)	18 (50%)	0.062
Virus	Influenza	1 (2%)	0 (0%)	0.36
	Parainfluenza	3 (7%)	0 (0%)	0.11
	Respiratory Syncytial virus	2 (5%)	0 (0%)	0.20
	Adenovirus	0 (0%)	1 (3%)	0.27
	Herpesviruses	1 (2%)	2 (6%)	0.44
	Human Metapneumovirus	0 (0%)	1 (3%)	0.27
	Enterovirus	0 (0%)	0 (0%)	NA

Other virus	0 (0%)	0 (0%)	NA
Sum of patients with viral coinfection	6 (14%)	4 (11%)	0.73
No bacterial, fungal, or viral organism identified	10 (23%)	15 (42%)	0.069

[a] p-value based on Two-sample Wilcoxon rank-sum (Mann-Whitney) test

[b] first observation, the measure must be obtained within the first 60minutes of PICU admission

ARDS, Acute Respiratory Distress Syndrome; ECMO, extracorporeal membrane oxygenation; PICU, paediatric intensive care unit; PIM2, paediatric index of mortality 2
