# Supplementary material for: Defining benefit threshold for extracorporeal membrane oxygenation in children with sepsis—a binational multicenter cohort study
Source: Crit Care. 2019 Dec 30;23:429. doi: 10.1186/s13054-019-2685-1 (PMC6937937; doi:10.1186/s13054-019-2685-1)

Additional File 5: Distribution of predicted mortality.

The predicted mortality risk is adjusted for covariates on respiratory failure (PaO2/FiO2 ratio, intubation, treatment with HFOV), cardiovascular (arterial hypotension, cardiac arrest pre ICU admission), metabolic (high lactate), Central Nervous System (dilated pupils), and renal (need for renal replacement) dysfunction, and underlying immunosuppression.

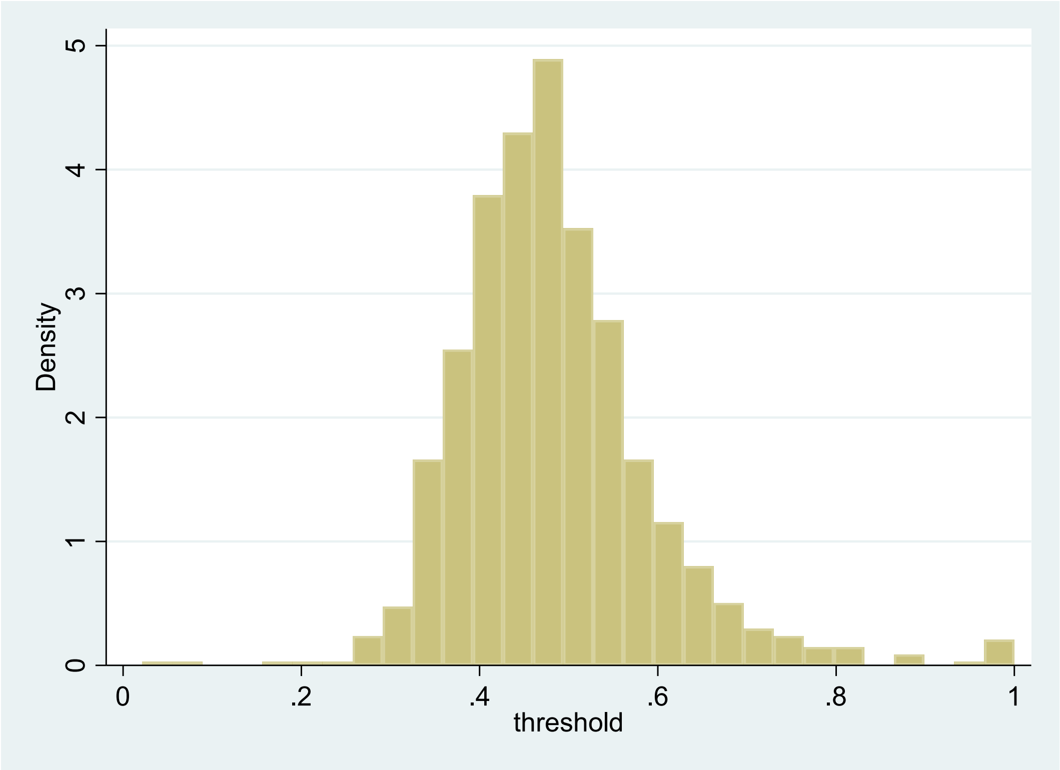

Supplement: Supplementary file 5 — Additional file 5. Distribution of predicted mortality. [file 13054_2019_2685_MOESM5_ESM.pdf]
