# Supplementary material for: Defining benefit threshold for extracorporeal membrane oxygenation in children with sepsis—a binational multicenter cohort study
Source: Crit Care. 2019 Dec 30;23:429. doi: 10.1186/s13054-019-2685-1 (PMC6937937; doi:10.1186/s13054-019-2685-1)
Supplement: Supplementary file 6 — Additional file 6. Sensitivity analysis to estimating treatment benefit for children with septic shock treated with Extracorporeal life support (ECMO, n=80) versus controls (n=2347), restricted to patients coded as septic shock in the ANZPIC registry. [file 13054_2019_2685_MOESM6_ESM.pdf]

Additional File 6: Sensitivity analysis to estimating treatment benefit for children with septic shock treated with Extracorporeal life support (ECMO, n=80) versus controls (n=2347), restricted to patients coded as septic shock in the ANZPIC registry. The marginal mean for estimated mortality is shown (y-scale) versus the baseline mortality score (x-scale) for children treated with ECMO (dark blue line) versus controls (light blue line). Full lines indicate the effect estimate, dashed lines indicate 95%-confidence intervals. The benefit threshold, defined as the baseline risk for which ECMO became beneficial reflects the intersection of both lines with is at 47.7% predicted risk of mortality (95% CI: 17.4%-93.6%). The predicted mortality risk is adjusted for covariates on respiratory failure (PaO2/FiO2 ratio, intubation, treatment with HFOV), cardiovascular (arterial hypotension, cardiac arrest pre ICU admission), metabolic (high lactate), Central Nervous System (dilated pupils), and renal (need for renal replacement) dysfunction, and underlying immunosuppression.

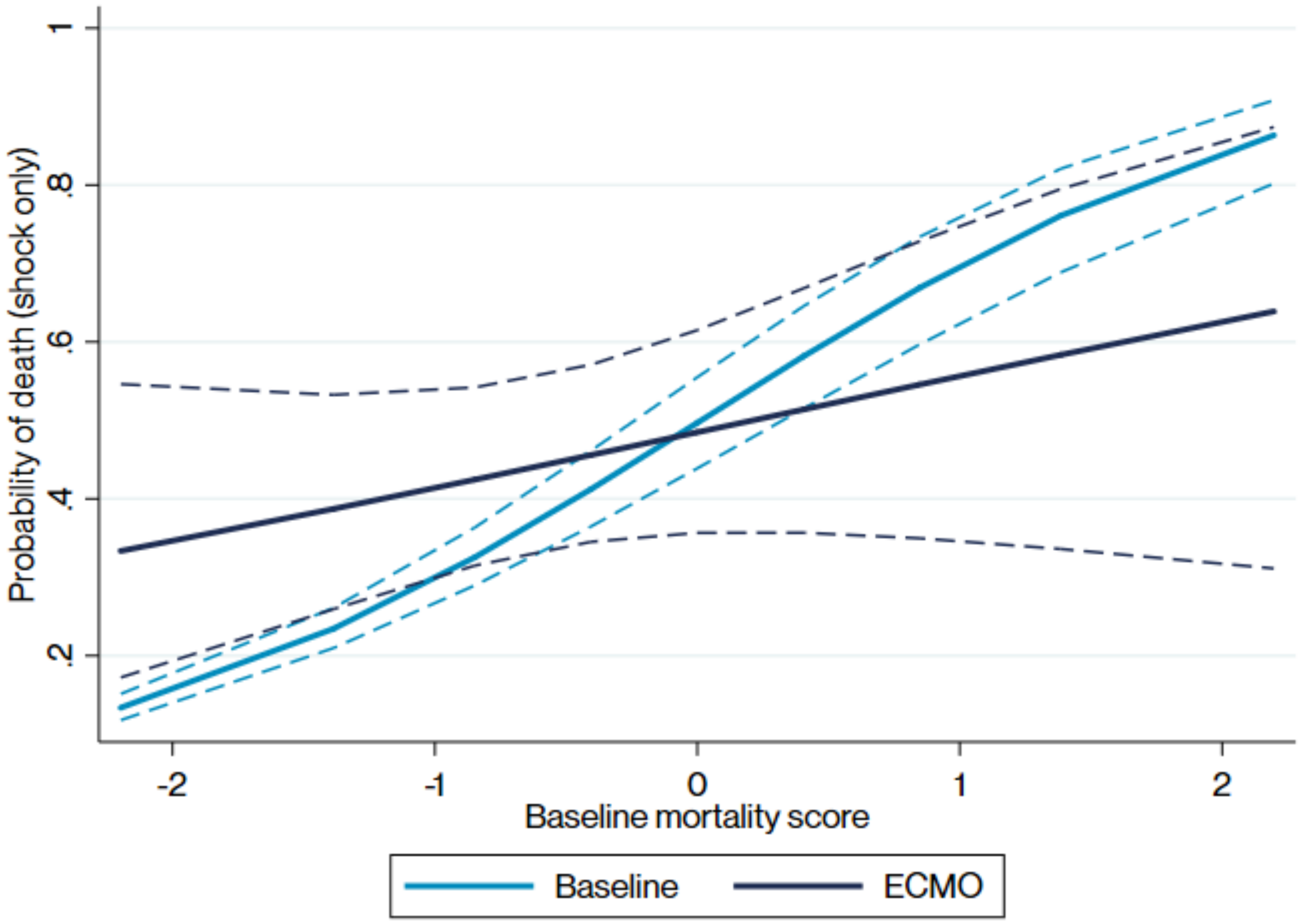

Probability of death (shock only)
.2
.4
.6
.8
1
-2
-1
0
1
2
Baseline mortality score
Baseline
ECMO
