# Supplementary material for: Defining benefit threshold for extracorporeal membrane oxygenation in children with sepsis—a binational multicenter cohort study
Source: Crit Care. 2019 Dec 30;23:429. doi: 10.1186/s13054-019-2685-1 (PMC6937937; doi:10.1186/s13054-019-2685-1)

Additional File 7: Extracorporeal Membrane Oxygenation (ECMO) flow rates measured at 4 hours (in ml/kg/min) post cannulation in n=80 children treated with veno-arterial ECMO for septic shock. Flow rates are compared between children that underwent peripheral cannulation (n=23) versus children that underwent central cannulation (n=57), split into those that survived (n=44) and that died (n=36).

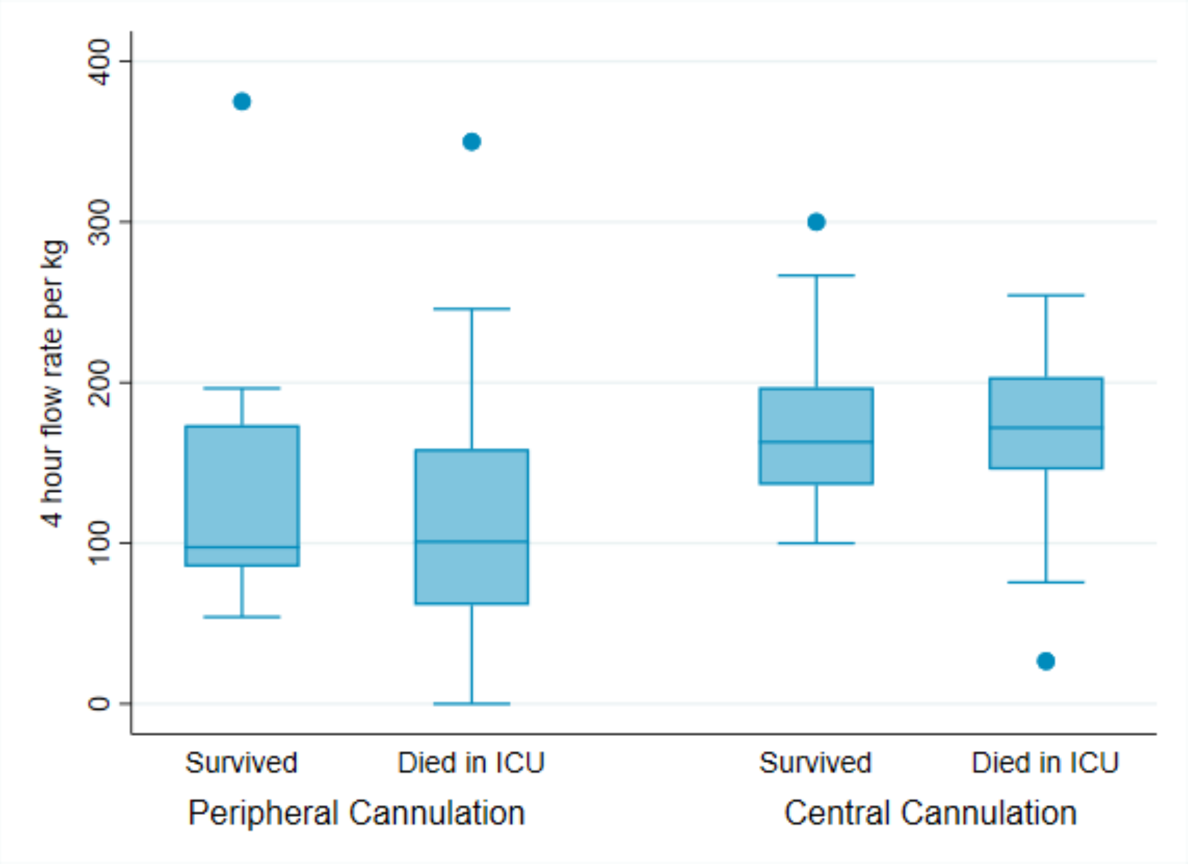

Supplement: Supplementary file 7 — Additional file 7. Extracorporeal Membrane Oxygenation (ECMO) flow rates measured at 4 hours (in ml/kg/min) post cannulation in n=80 children treated with veno-arterial ECMO for septic shock. Flow rates are compared between children that underwent peripheral cannulation (n=23) versus children that underwent central cannulation (n=57), split into those that survived (n=44) and that died (n=36). [file 13054_2019_2685_MOESM7_ESM.pdf]
